# Estimation of viral richness from shotgun metagenomes using a frequency count approach

**DOI:** 10.1186/2049-2618-1-5

**Published:** 2013-02-04

**Authors:** Heather K Allen, John Bunge, James A Foster, Darrell O Bayles, Thaddeus B Stanton

**Affiliations:** 1 Food Safety and Enteric Pathogens Research Unit, National Animal Disease Center, Agricultural Research Service, Ames, IA, 50010, USA; 2 Department of Statistical Science, Cornell University, Ithaca, NY, 14583, USA; 3 Department of Biological Sciences, Institute for Bioinformatics and Evolutionary Studies, University of Idaho, Moscow, ID, 83844, USA; 4 Infectious Bacterial Diseases Research Unit, National Animal Disease Center, Agricultural Research Service, Ames, IA, 50010, USA

**Keywords:** Phage, Metagenomics, Virome, Ecology, Richness, CatchAll, Singleton

## Abstract

**Background:**

Viruses are important drivers of ecosystem functions, yet little is known about the vast majority of viruses. Viral shotgun metagenomics enables the investigation of broad ecological questions in phage communities. One ecological characteristic is species richness, which is the number of different species in a community. Viruses do not have a phylogenetic marker analogous to the bacterial 16S rRNA gene with which to estimate richness, and so contig spectra are employed to measure the number of virus taxa in a given community. A contig spectrum is generated from a viral shotgun metagenome by assembling the random sequence reads into groups of sequences that overlap (contigs) and counting the number of sequences that group within each contig. Current tools available to analyze contig spectra to estimate phage richness are limited by relying on rank-abundance data.

**Results:**

We present statistical estimates of virus richness from contig spectra. The program CatchAll (http://www.northeastern.edu/catchall/) was used to analyze contig spectra in terms of frequency count data rather than rank-abundance, thus enabling formal statistical analyses. Also, the influence of potentially spurious low-frequency counts on richness estimates was minimized by two methods, empirical and statistical. The results show greater estimates of viral richness than previous calculations in nearly all environments analyzed, including swine feces and reclaimed fresh water.

**Conclusions:**

CatchAll yielded consistent estimates of richness across viral metagenomes from the same or similar environments. Additionally, analysis of pooled viral metagenomes from different environments via mixed contig spectra resulted in greater richness estimates than those of the component metagenomes. Using CatchAll to analyze contig spectra will improve estimations of richness from viral shotgun metagenomes, particularly from large datasets, by providing statistical measures of richness.

## Background

Viruses are the most abundant biological entities on earth, with an estimated 10^31^ virus-like particles in the biosphere [1]. Their ubiquity coupled with their functions of predation and gene transfer make them important drivers of ecosystem dynamics, as illustrated during cholera outbreaks. When a cholera outbreak strikes, the abundance of the causative bacterium, *Vibrio cholerae*, is high. Bacteriophages (phages) that prey on the *V. cholerae* then proliferate, and the outbreak subsides as the abundance of *V. cholerae* declines due to phage predation [2,3].

These dynamics are constantly played out in the environment with non-pathogenic bacteria and their phages. However, even though plaque and culture assays remain the gold standard for studying the phages of a cultivable bacterium, the vast majority of environmental bacteria have yet to be cultured [4]. Therefore, viral shotgun metagenomics, which is the study of the collective genome of an assemblage of viruses, is the principal way to study the vast majority of phages. Next-generation sequencing technologies are essential to study phage metagenomes and phage ecology.

A first step toward understanding the complex interactions that occur in an environment is estimating the richness of species in that environment. Richness is the total number of distinct members in a community and, with the abundance of each member, contributes to the total diversity. The distinct members are often measured in terms of species, but because viruses lack a species definition we will refer to distinct groups of viruses as taxa. Viruses do not have a universal phylogenetic marker analogous to the bacterial 16S rRNA gene with which to measure richness, and so contig spectra serve as a proxy to estimate the number of phage taxa in a given community. A contig spectrum is generated from a viral metagenome, or virome, by assembling the random sequence reads into contigs (contiguous groups of sequences that align) and counting the number of sequences that fall into each contig [5,6]. The rationale is that in any given sampling of an environment, abundant viruses will yield many sequences in one contig, whereas rare viruses will be captured as single sequences. Counting the sequences in terms of their assembly proficiency, therefore, reflects the richness of the community.

**Figure 1:**
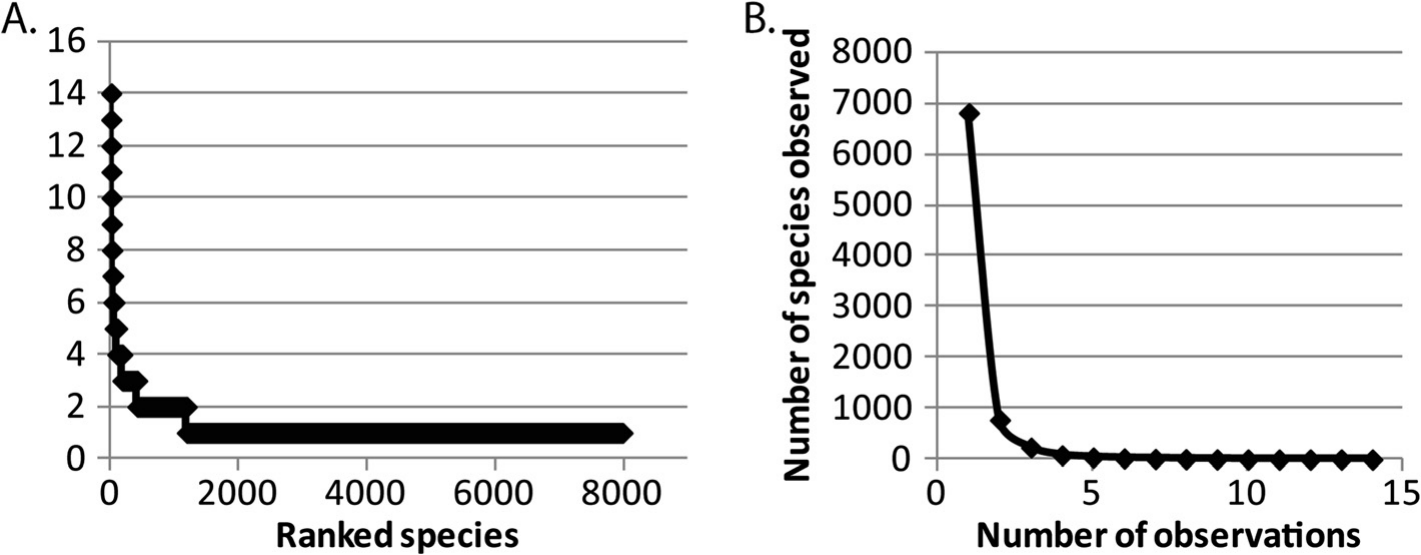
**Rank abundance versus frequency count data plots.** The contig spectrum of a swine phage metagenome (RL1.NonmedDay0, [7]) was graphed by rank abundance (**A**) or frequency count (**B**) methods to illustrate the difference in representation of the same data.

The program PHACCS (**Pha**ge **C**ommunities from **C**ontig **S**pectrum) was developed to estimate the richness and evenness of phage taxa based on their contig spectra [1]. However, the richness computation relies on rank-abundance curves instead of frequency count data. There is a subtle but crucial distinction between the rank abundance curve and the frequency count curve. Both begin with a sample (of organisms, sequences or some kind of signature) that is binned into groups (such as bacterial species or phage taxa), and the sizes of the groups are recorded. For the rank-abundance curve, the observed species are then sorted from most to least numerous, and the resulting data are graphed. The most-sampled species is plotted leftmost, the next most-sampled species next, and so on, leading to a large number of singletons trailing off to the right (Figure 1A). This is a qualitative, not a quantitative, representation of species abundance. For example, every rank-abundance curve will be monotonically decreasing (from left to right), even though the actual occurrences of the species in the sample are random, and the most common species in the sample may not be the most common in the population.

In the frequency-count approach, by contrast, a fixed x-axis is established and the number of species observed x times is plotted as the y-value for each x (Figure 1B). This apparently simple distinction has major consequences. In the rank-abundance curve, both the x and the y-axes are indeterminate, because the ordering of the species in terms of their true abundance in the population can be very different from the observed ordering. A fixed x-axis yields a dataset that is amenable to formal statistical analysis, which is important because the value of y at x = 0 (the number of unobserved species) is the target of estimation.

Here we apply the analysis of frequency count data to previously published phage metagenomes. We present statistical estimates of richness from phage metagenomic data using the program CatchAll version 3.0 (http://www.northeastern.edu/catchall/index.html) [8]. Additionally, we explore the effect of statistically and empirically discounted low-frequency datapoints on the richness estimates. Even the most conservative richness estimates show more phage taxa than previous calculations, in most environments analyzed.

## Methods

### Generating contig spectra

Both mixed and non-mixed contig spectra of seven nonmedicated swine fecal viromes [7] and four reclaimed fresh water viromes [9] were calculated using Circonspect (http://sourceforge.net/projects/circonspect/) [6]. Artificial replicates were removed from the viromes prior to the analysis [10]. Other viromes (salt water [6,11,12], human infant fecal [11], and human adult fecal [12]) were analyzed based on their published contig spectra. All contig spectra used in this study are reported (Additional file 1). Circonspect generates contig spectra based on the assembly of the user’s viral metagenome(s). The default assembler (Minimo; http://sourceforge.net/apps/mediawiki/amos/index.php?title=Minimo) was employed [13,14]. Settings that can be adjusted by the user include the maximum sequence length at which to trim the input sequences (trim), the shortest sequence length to allow in the assembly (discard), the number of sequences to sample (sample size), and the depth of coverage to target. The input settings were optimized to maximize the assembled data used in calculating the contig spectra and to minimize the error: trim length, 600 (that is, greater than the average read length of the sequences in the sample); discard length, 100; sample size, 10,000 sequences per metagenome; coverage, 2x (that is, enough times to query each read twice). Settings not listed were not changed from default. These settings are comparable to what have been used previously [15] and fit within the current computation limits of Circonspect.

### Estimating richness from contig spectra

All contig spectra were then analyzed in CatchAll and PHACCS [1], the latter of which was implemented via CAMERA’s alpha diversity pipeline (http://camera.calit2.net/) [16]. Data were loaded in comma-delimited files into CatchAll version 3.0 [8], which estimates the richness using parametric and nonparametric models. CatchAll postulates a flexible family of stochastic abundance models, and fits or estimates these by maximum likelihood (via sophisticated numerical search algorithms; see [8,17] for a discussion of the different models calculated by CatchAll). This procedure is known to be optimal when the postulated abundance model is indeed the true model. Unfortunately, it is not possible to know what the true model is, although the issue can be addressed via modern goodness-of-fit analysis as performed by CatchAll. This uncertainty is addressed by the flexibility of the models used by CatchAll, leading the statistical analyses to be moderately robust. To address further departures from the postulated parametric models, or certain other violations of assumptions, CatchAll also implements nonparametric estimation methods. These attempts to make minimal assumptions about the underlying population structure broaden the model base, but in turn exact a price in terms of statistical efficiency, that is, the variance of the final estimate per unit of sample size. (These issues have been well explored in the theoretical statistics literature.) In short, CatchAll fits a suite of flexible parametric models, along with a suite of nonparametric analyses, compares these and returns the best results according to statistical and heuristic criteria. Additionally, CatchAll performs statistical discounting of low-frequency observations and optionally provides the discounted richness estimate, as reported previously [8,18].

### Empirical discounting of contig spectra

MG-RAST (http://metagenomics.anl.gov/) [19] was used to assign the singleton sequence reads of three swine viromes to a taxonomic origin (bacteria, viral, eukaryote, archaeon, unassigned or no hits). Previous results showed that these viromes contained almost entirely phage DNA [7]. Because of the inability to annotate the vast majority of phage genes, the real phage sequences might have no hits or would be assigned to viruses, while sequences in the other categories would be spurious. Assigning the singletons and tallying the number of spurious assignments lacks precision because even specialized phages are known to carry bacterial genes [20], but it is nonetheless a reasonable point of reference to help gauge the discounted richness estimates. Under these assumptions, the ratio of real to spurious reads was calculated, ranging from 1:1 to 1:2. The numbers of singletons were, therefore, halved within each contig spectrum to manually dispose of the supposedly spurious reads, and the resulting spectra were analyzed in CatchAll as above.

## Results and discussion

### Richness estimates of published phage metagenomes

We applied current statistical procedures [8,17,18] to calculate richness from individual viral metagenomes. The results show high estimates of viral richness under the best parametric model in CatchAll (Table 1; Chao-type nonparametric estimates, such as ACE (not shown here) are roughly comparable to the best parametric results; note that the PHACCS estimates do not provide standard errors). The CatchAll richness estimates ranged from one to three orders of magnitude higher than the PHACCS estimates, suggesting that previous reports have underestimated viral richness in all environments analyzed (Table 1). Additionally, CatchAll richness estimates tended to be more consistent across viral metagenomes of the same or similar environments. The salt water viromes appear to deviate from this trend, but the inputs for the metagenomes were different: British Columbia and Gulf of Mexico viromes actually represent numerous samples over time, whereas the Sargasso Sea virome originated from a single sample.

**Table 1:** Comparison of richness estimates for published viral metagenomes

**Virome**	**CatchAll richness estimate**			**PHACCS richness estimate**	**Virome**
	**Best parametric model ± SE**^**a**^			estimate	
	***All singletons***	***Empirically discounted***	***Statistically discounted***	***Power law model***	**Reference**
*Mammalian gut environments*					
Nonmedicated swine feces, 21 d^b^	90,576 ± 7,717	20,781 ± 1,054	2,381 ± 203	360	[7]
Nonmedicated swine feces, 35 d	124,284 ± 11,985	17,581 ± 762	9,693 ± 935	405	[7]
Nonmedicated swine feces, 38 d	84,524 ± 23,415	14,663 ± 592	4,686 ± 1,298	246	[7]
Nonmedicated swine feces, 63 d	105,310 ± 48,167	16,267 ± 2,190	5,362 ± 2,452	164	[7]
Nonmedicated swine feces, 77 d	130,773 ± 44,679	22,879 ± 3,381	5,071 ± 1,733	357	[7]
Nonmedicated swine feces, 85 d	113,335 ± 7,958	27,650 ± 1,478	1,307 ± 92	787	[7]
Nonmedicated swine feces, 91 d	154,869 ± 59,005	24,202 ± 1,139	5,386 ± 2,052	703	[7]
Human infant feces^c^	1,087 ± 348	344 ± 74	94 ± 30	8	[11]
Human adult feces^c^	9,576 ± 1,810	2,733 ± 517	NA^d^	1,930	[12]
*Aquatic environments*					
Reclaimed fresh water, potable	59,741 ± 5,150	14,259 ± 803	2,388 ± 206	184	[9]
Reclaimed fresh water, effluent	128,778 ± 10,752	29,882 ± 1,833	1,617 ± 135	764	[9]
Reclaimed fresh water, nursery	204,571 ± 75,474	37,260 ± 7,320	4,477 ± 1,652	1,754	[9]
Reclaimed fresh water, park	185,739 ± 15,756	42,854 ± 2,899	1,043 ± 88	98,603	[9]
Salt water, Gulf of Mexico^c^	246,019 ± 90,045	59,696 ± 21,341	103 ± 37	15,400	[6]
Salt water, British Columbia^c^	320,708 ± 73,575	81,644 ± 18,730	NA	129,000	[6]
Salt water, Sargasso Sea^c^	108,264 ± 14,870	28,701 ± 3,942	NA	5,140	[6]
Salt water, Arctic Ocean^c^	NA	NA	NA	532	[6]
*Pooled viromes*					
The seven swine fecal viromes	155,469 ± 16,052	34,512 ± 2,360	1,990 ± 206	ND^e^	[7]
The four reclaimed fresh water viromes	183,920 ± 18,009	41,751 ± 3,284	1,428 ± 140	ND	[9]
Nonmedicated swine feces, 85 d, mixed with reclaimed fresh water, effluent	196,069 ± 23,490	43,205 ± 3,865	1,958 ± 235	ND	[7,9]
The four saltwater viromes^c^	668,901 ± 269,866	151,974 ± 54,948	1,272 ± 513	57, 600 [16]	[6]

^a^SE, standard error.

^b^d, days old.

^c^The contig spectra published in the corresponding reference were run in CatchAll.

^d^NA, not available because the contig spectra did not contain enough data to perform the calculation.

^e^ND, not determined.

In some cases the sample is so small that it becomes impossible to estimate richness. For example, the contig spectrum of some phage metagenomes, such as the Arctic Ocean salt water virome [6], lacked sufficient data for the richness to be calculated by CatchAll (Table 1). As the cost of sequencing continues to decline, large virome datasets amenable to the present analysis should become more plentiful. This also should encourage the sequencing of biological replicates to enable statistical comparisons between metagenomic datasets.

### Estimated richness after statistical discounting

Although the CatchAll-based richness estimates were roughly on the same order of magnitude per environment, we reasoned that 100,000 viral taxa per sample could be an overestimate of the true richness. This is because contig spectra from viral metagenomes might contain a large number of spurious singletons, due to both biological and technical phenomena. An example of a biological phenomenon is that certain viruses of bacteria (bacteriophages or phages) called generalized transducing phages are known to package random pieces of bacterial chromosomal DNA, which would never assemble in a contig spectrum and thereby inflate the number of singletons. Another biological phenomenon that could inflate the richness estimates is the mosaicism of phage genomes [21], which could decrease the assembly of related phages and cause them to be counted as discrete taxa. From a technical standpoint, pyrosequencing is error prone: errors introduced by pyrosequencing technology inflate 16S rRNA-based estimates of diversity [22], and data derived from bulk DNA sequencing need to be screened for false duplicates that arise from the emulsion PCR step [10].

Ideally, any suspected inflated diversity would be fixed at an appropriate technical step in the sequencing pipeline. In the absence of a technical solution, we theoretically discussed several statistical discounting procedures [18] and modified CatchAll to optionally implement a statistical procedure that discounts the low-frequency observations, such as singletons [8]. The results of statistically discounting the present contig spectra yielded phage richness estimates in the thousands per sample, which may be overly strict but yields a more biologically intuitive result than hundreds of thousands of phage taxa per sample (Table 1). This is the first broad application of this technique to multiple datasets.

### Estimated richness after empirical discounting

Our statistical discounting procedure deletes a proportion of the low-frequency observations at the data analysis stage rather than data production stage, yielding what may be an overly conservative richness estimate. When juxtaposed with the high-richness estimates based on the original data, there is an expansive difference between the original unadjusted estimate and discounted richness estimates for a virome (Table 1). How, then, do we reconcile these estimates with the biology? An ideal proof-of-principle for the discounting procedure would be to compare the richness estimates to a scenario in which we actually know how many low-frequency counts are spurious. To address this, we developed an empirical discounting method for three swine phage metagenomes using the taxonomic assignment of the reads to infer a ratio of real to spurious reads. This ratio suggested that half of the reads could be spurious, and so the number of singletons in the contig spectra were halved prior to estimating the richness in CatchAll. The new estimated phage diversities were approximately 20% of the estimates based on the original contig spectra, but still 5 to 35 times greater than the statistically discounted estimates (Table 1). We, therefore, conclude that the statistical discounting method is indeed more conservative than inferring a discount based on the taxonomic information in the singletons. These empirical results also suggest that analyses of contig spectra that include potentially spurious reads at least double the richness estimate. The mathematically discounted model is a statistically sound tool to estimate the minimum richness of large viral metagenomic datasets.

### Comparing phage richness between environments

When comparing phage metagenomes from different environments via mixed contig spectra from pooled viromes, CatchAll nearly recovers the expected reality that the summed richness is greater than that of the member environments. A mixed contig spectrum is achieved by mixing the sequences from two or more environments prior to generating a contig spectrum [6]. We analyzed a published mixed contig spectrum from four salt water viromes [6] in CatchAll, resulting in a combined phage richness that was nearly equivalent to the sum of the richness of three component environments (Table 1). This result contrasted with the combined phage richness estimated by PHACCS, which showed fewer phage taxa in the mixed environments than in one of the component environments, indicating that the majority of phage taxa could be shared among environments [6].

We further explored this phenomenon by generating a mixed contig spectrum for seven non-medicated swine viromes, and again for four reclaimed water viromes. The mixed swine viromes showed an estimated richness just greater than the most rich component virome (155,468 ± 16,052; Table 1). The individual swine viromes were generated from the same six pigs over time, and so it is likely that a large part of the community would be shared among the viromes. This is reflected in the pooled-virome richness estimate being one-fifth of the sum of the richness of the component environments, and in the substantially decreased standard error of the richness estimate resulting from the increased sample size. The reclaimed water viromes are similarly related in that they all originated from the same wastewater treatment facility, and the mixed spectra richness estimate showed a similar trend (Table 1). Note that this trend does not hold when examining the statistically discounted richness estimates. This is because the high-diversity component is larger in the pooled sample, and so its removal after statistical estimation has a larger impact on the richness estimate than either its inclusion (no discounting) or prior removal during empirical discounting.

To test the effect of pooling two very different environments, we mixed one swine virome (Day 85) with one reclaimed water virome (effluent). The estimated richness (196,069) was about 40% greater than the richness of either component environment. Importantly, the standard error (±23,489) was also greater, suggesting that unlike in the mixed analyses of similar environments, the depth of coverage was not improved by pooling unlike samples. Our results suggest that the majority of inter-environmental phage taxa are not shared. This is aligned with current dogma for microbial biogeography indicating that both viral taxa and bacterial species are heterogeneously distributed based on habitat and spatial structure [23,24].

It is tempting to compare the differences between the estimated numbers of phage taxa per environment in Table 1, such as noting that the discounted richness estimates for swine fecal viromes are roughly double the discounted richness estimates of reclaimed fresh water. However, it is impossible to draw conclusions because of the lack of depth and repetition of any given data set. Expanding the breadth and depth of phage metagenomic studies will improve inter-environmental comparisons and thus advance biological conclusions.

## Conclusions

The statistical diversity estimation procedures implemented in CatchAll improve upon comparable previous implementations. The accuracy of any estimates of viral richness is unknown because the sampling of nature is so very, very sparse. Tests of accuracy in given communities with known diversity, or in simulated ones, can provide some perspective, but such results are not generalizable beyond the specific cases studied, and hence are limited. Instead, statisticians turn to general theoretical optimality principles, which underlie the numerous procedures employed by CatchAll to perform both parametric and nonparametric analyses. An additional improvement is that CatchAll provides confidence intervals that bound the uncertainty within the limits of the available data.

In addition to a sound statistical foundation, we employed discounting approaches to investigate the effect of potentially spurious low-frequency counts on richness estimates. The theory behind the statistically discounted approach was presented elsewhere [18], but this is its first application to multiple datasets. The empirically discounted estimates are new to the present manuscript. Discounting provides an option for estimating richness from samples that are suspected to contain spurious low-frequency observations. Further studies are needed to elucidate the effect of biological features, such as genetic mosaicism, on estimates of phage richness.

The non-discounted richness estimates reveal more viral species per environment than previous metagenomic-based estimates, and also greater consistency in the estimates between like environments. Additionally, analysis of pooled viromes from disparate environments showed the expected result: mixing increased both the richness estimate and the error associated with that richness. Increased depth of sequencing coverage will improve the accuracy of richness estimates, and technologies are quickly advancing to enable deep metagenomic sequencing. Improved richness estimates should dramatically improve the inferences possible in phage ecological studies.

## Abbreviations

PHACCs: Phage Communities from Contig Spectrum.

## Competing interests

The authors declare that they have no competing interests.

## Authors’ contributions

HKA analyzed data and drafted the manuscript. JAB conceptualized the new method and designed and performed analyses. JAF contributed to the development of the analysis. DOB performed analyses. TBS provided oversight of the work. All authors read and approved the final manuscript.

## Supplementary Material

Additional file 1 Contig spectra used in this study. Click here for file
